# Is gallbladder inflammation more severe in male patients presenting with acute cholecystitis?

**DOI:** 10.1186/s12893-015-0034-0

**Published:** 2015-04-24

**Authors:** Peter C Ambe, Sebastian A Weber, Dirk Wassenberg

**Affiliations:** Department of General, Visceral and thoracic surgery, St. Remigius Hospital Opladen, An St. Remigius 26, 51379 Leverkusen, Germany; Department of Internal Medicine, St. Elisabeth Hospital Hohenlind, Werthmannstr. 1, 50937 Köln, Germany; Helios Klinikum Wuppertal, Department of Surgery II, Witten - Herdecke University, Heusner Str. 40, 42283 Wuppertal, Germany

**Keywords:** Acute cholecystitis, Laparoscopic cholecystectomy, Male gender, Gangrenous cholecystitis, Necrotizing cholecystitis

## Abstract

**Background:**

The male gender is considered a risk factor for complications in patients undergoing laparoscopic cholecystectomy. The reasons for this gender associated risk are not clearly understood. The extent of gallbladder inflammation has been shown to influence surgical outcome. The aim of this study was to investigate whether or not gallbladder inflammation is more severe in male patients presenting with acute cholecystitis.

**Methods:**

A retrospective gender dependent comparison of the data of patients undergoing laparoscopic cholecystectomy for acute cholecystitis in a primary care facility within a five-year period was performed.

**Results:**

138 patients, 69 males and 69 females were included for analysis. Severe gallbladder inflammation (gangrenous and necrotizing cholecystitis) was seen in a significant portion of the male population compared to the female population (p = 0.002). The male gender was confirmed in a multivariate analysis as an independent risk factor for severe cholecystits (p = 0.018).

**Conclusion:**

The male gender is a risk factor for severe gallbladder inflammation. An early surgical intervention may be needed to prevent complications.

## Background

Laparoscopic cholecystectomy (LC) for acute cholecystitis can be challenging and surgical outcome seems to be greatly influenced by the extent of inflammation [1-3]. Higher rates of complications have been reported in patients undergoing laparoscopic cholecystectomy for gangrenous and empyematous cholecystitis [4-8]. Equally, the male gender has been identified as an independent risk factor for complications in patients undergoing LC, both for symptomatic cholecystolithiasis and for acute cholecystitis [9-13]. The aim of this study was to verify whether or not gallbladder inflammation is more severe in male patients undergoing LC for acute cholecystitis (AC).

## Methods

Following the approval of the ethics committee at the Saint Remigius Hospital Opladen, a retrospective review of the charts of patients undergoing LC for AC was performed. Using the ICD 10 codes for acute cholecystitis, a search of our institutional database for patients undergoing surgery for AC from the January 2009 to December 2013 was performed. Baseline data including sex, age, body mass index (BMI) and comorbidity score as defined by the American Society of Anesthesiologists (ASA) were recorded for each patient.

Acute Cholecystitis was diagnosed as recommended in the Tokio guidelines [14,15]. Only patients managed with LC within 72 hours following admission were included for analysis. Surgery was performed by an experienced surgical attending. Four incisions were used in all cases. Pneumoperitoneum was installed via an infra-umbilical mini-laparotomy and the maximum intraabdominal pressure was set at 12 mmHg. Information on intraoperative blood loss was retrieved from the anesthesiology protocol while postoperative complications and the length of stay were retrieved from the final discharge documents. The extent of gallbladder inflammation was retrieved from the final histopathology reports.

The data acquired was analyzed using the Statistical Package for Social Sciences, SPSS, IBM, Version 22. The study population was statistically described using absolute number of cases and percentages. Central tendencies were described using median values and interquartile ranges, while significances were calculated using the 2 × 2 chi square test and reported as significant with p < 0.05. Outcomes of the male population were compared to those of the female population.

## Results

**Figure 1:**
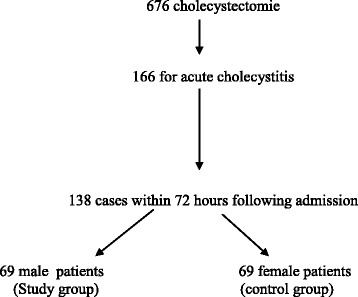
Distribution of the study population. 69 male patients were compared to 69 female patients undergoing laparoscopic cholecystectomy for acute cholecystitis.

Within the period of investigation, 676 cases of cholecystectomy were recorded. The indication for surgery was AC in 166 cases. One hundred and thirty-eight cases of LC for AC were performed within 72 hours of admission, Figure 1. A summary of the baseline characteristics of the study population is presented in Table 1. Both groups were comparable with regard to baseline characteristics.

**Table 1:** **Summary of the baseline features of the study population**

**Features**	**Female group**	**Male group**	**p- value**
Number of cases	69	69	/
Median age (yrs)	66.0 yrs	72.0 yrs	0.20
Interquartile range	25.0 yrs	17.0 yrs	
Median BMI (kg/m^2^)	28.7	27.6	0.40
Interquartile range	9.3	7.5	
ASA 1-2	44 (63.8%)	36 (52.2%)	0.26
3-4	25 (36.2%)	33 (47.8%)	

Yrs: years.

There was no significant difference amongst both groups with regard to white blood count (14.6/ul vs. 14.1/ul for the female and male group respectively, p = 0.56) and c - reactive protein (17.1 mg/dl vs. 20.0 mg/dl for the female and male group respectively, p = 0.15).

Extensive gallbladder inflammation in the form of necrotizing or gangrenous cholecystitis was recorded in a significant number of male patients compared to female patients (43/69 vs. 21/69, p = 0.002). A multivariate analysis identified the male gender as an independent risk factor for severe gallbladder inflammation, Wilk’s λ = 0.94, F (2, 135) = 4.14, p = 0.018, partial η^2^ = 0.06. Table 2 summarizes the severity grades and the extent of gallbladder inflammation (on histopathology) in both groups.

**Table 2:** **Summary of the perioperative data of the study population**

**Features**	**Female group**	**Male group**	**p -value**
Severity grade			0.91
- I	39 (56.5%)	40 (58.0%)	
- II	18 (26.1%)	15 (21.7%)	
- III	12 (17.4%)	14 (20.3%)	
Extent of inflammation			0.002
- Edematous	48 (69.6%)	26 (37.7%)	
- gangrenous	14 (20.3%)	20 (29.0%)	
- necrotizing	7 (10.1%)	23 (33.3%)	
Median intraop. blood loss	180 ml	250 ml	0.04
Interquartile range	60 ml	110 ml	

*Necrotizing cholecystitis: total necrosis of all layers of gallbladder wall with suppurative inflammation and surface erosion.*

*Gangrenous cholecystitis: loss of mucosal lining and vascular architecture with profuse inflammation secondary to focal gallbladder ischemia.*

The median length of anesthesia was 121 minutes in the female and 126 minutes in the male group. This difference was not statistically significant (p = 0.23). Equally, there was no significant difference amongst both groups with respect to the duration of surgery (70 min vs. 75 min in the female and male group respectively, p = 0.13).

The rate of conversion in this series was 13.8% (19 cases). This corresponded to 11.6% (8 cases) in the female cohort and 15.9% (11 cases) in the male cohort. There was no significant difference amongst both groups with regard to the rate of conversion (p = 0.62). Complications were recorded in 23 cases (16.6%). The morbidity rate in the female group was 17.3% (12 cases). The following complications were recorded in the female group: three cases of wound infection, two cases of cystic duct leak and pulmonary embolism, one case of urinary tract infection, acute renal failure, pleura effusion, pneumonia and liver abscess. The rate of morbidity in the male cohort was 15.9% (11 cases) including four patients with cystic duct leak, three patients with wound infection, three patients with pneumonia and one patient with urinary tract infection. This difference was not statistically significant, p = 0.82. The median length of postoperative hospital stay was 6 days in both groups (p = 0.25). The mortality rate in this study was 1.4% (two cases). Both cases were recorded in the female cohort (2.8%) following pulmonary embolism and respiratory insufficiency. This finding was not statistically significant, p = 0.49.

## Discussion

This study was designed to investigate whether or not the extent of gallbladder inflammation is more severe in male patients presenting with acute cholecystitis. Sixty-nine male patients were compared with 69 female patients undergoing laparoscopic cholecystectomy for acute cholecystitis. Both groups were comparable with regard to perioperative characteristics. Equally, there was no difference amongst both groups with respect to post-operative complications. However, extensive gallbladder inflammation in the form of gangrenous and necrotizing cholecystitis was evident in a significant majority of the male population on histopathology compared to the female population. Multivariate analysis confirmed the male gender as an independent risk factor for extensive gallbladder inflammation.

The management of patients with AC could be associated with high rates of morbidity and mortality [16,17]. Surgical outcomes may be influenced by quiet a number of factors. The extent of gallbladder inflammation has been shown to affect the rate of complications in patients undergoing LC for acute cholecystitis [6,7,11]. According to G. Croley [18] severe inflammation of the gallbladder as seen in cases of gangrenous and necrotizing cholecystitis occurs as a result of vascular compromise following sustained cystic duct obstruction. The extensive inflammatory changes in such cases are associated with significantly higher rates of morbidity when compared with uncomplicated cholecystitis [19].

Available data suggests the male gender as a risk factor for complicated LC [10,13]. However, it is not known whether or not the risk associated with the management of male patients with acute cholecystitis is secondary to the male gender per se or to the extent of gallbladder inflammation.

The differences in the extent of gallbladder inflammation seen in this study cannot be blamed on age and comorbidities since both groups were comparable with regard to age and comorbidities. This is also true for the timing of surgery since all patients were managed within the same time frame (72 h). Furthermore, there was no risk of selection bias since all patients included were consecutively recorded.

The reasons for this gender associated difference are not well understood. However, male patients may have a higher threshold for pain. It is therefore thinkable that male patients might have experienced many episodes of undiagnosed cholecystitis which might have predispose to a more severe form of inflammation.

Taken together, our results suggest the male gender to be a risk factor for severe cholecystitis. Extensive gallbladder inflammation in the form of gangrenous and necrotizing cholecystitis was evident in a significant majority of male patients compared to female patients. Although there was a statistically significant difference in the median intraoperative blood loss amongst both groups, the absolute difference was just 70 ml. Since its clinical relevance is questionable, this finding must be interpreted with caution.

The trend presented in this series should appeal for an early surgical intervention in male patients with acute cholecystitis in order to prevent possible complications due to the extensive nature of gallbladder inflammation in this subgroup.

This study is limited by its retrospective design and the small size of the study population. Therefore the trend showed in this series warrants further investigation in a prospective set-up with well designed protocols and larger patient numbers*.*

## Conclusion

The male gender is a risk factor for severe gallbladder inflammation. An early surgical intervention may be needed to prevent complications.
